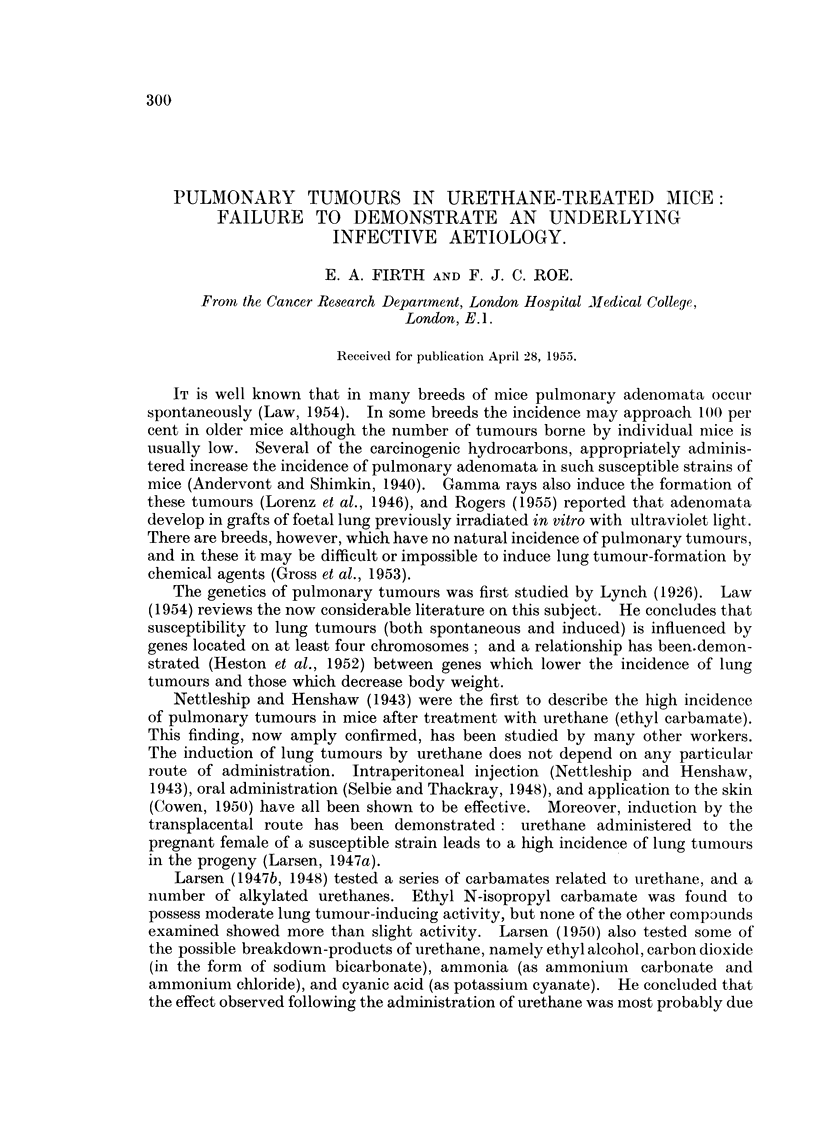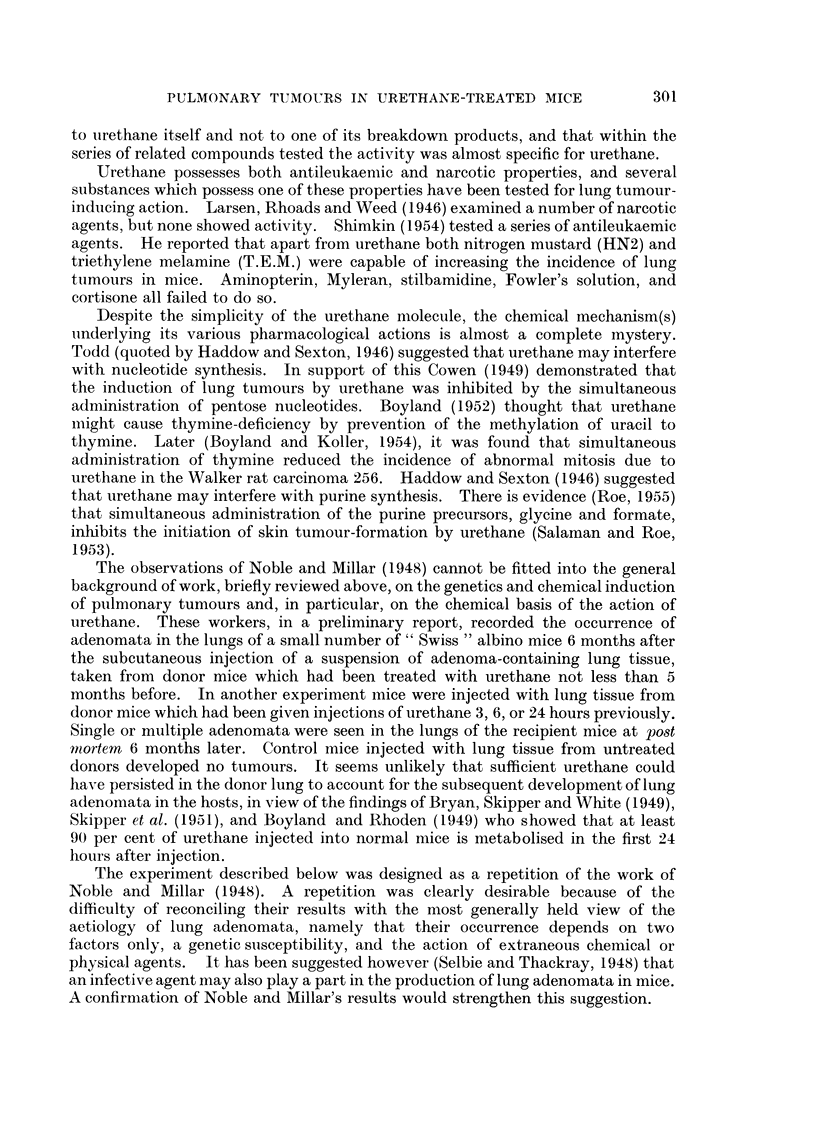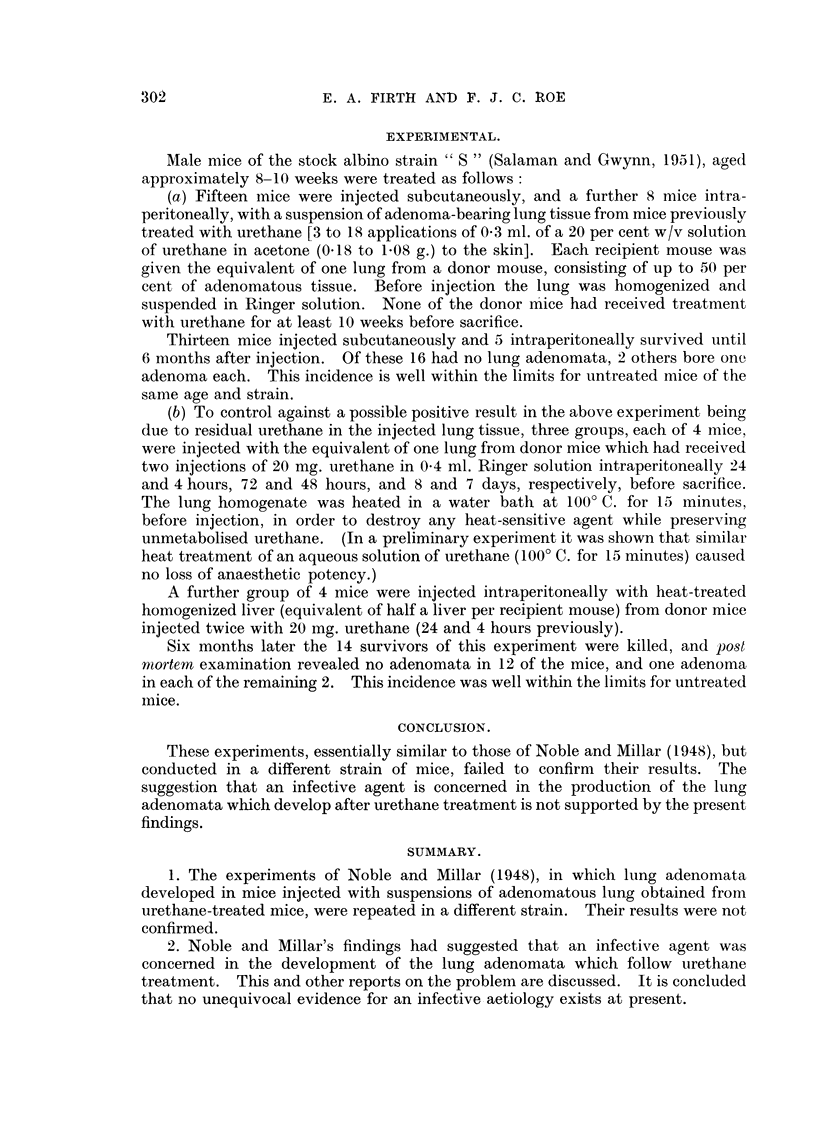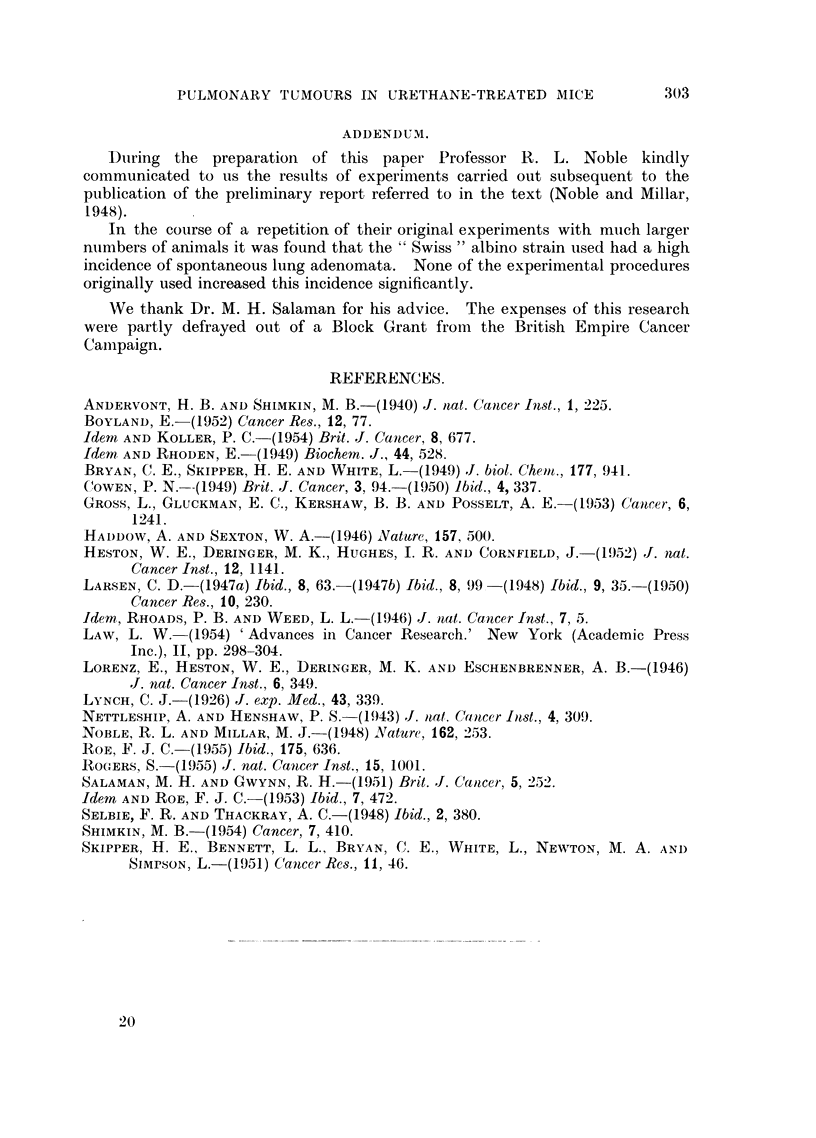# Pulmonary Tumours in Urethane-Treated Mice: Failure to Demonstrate an Underlying Infective Aetiology

**DOI:** 10.1038/bjc.1955.26

**Published:** 1955-06

**Authors:** E. A. Firth, F. J. C. Roe


### PULMONARY TUMOURS IN URETHANE-TREATED MICE: FAILURE TO DEMONSTRATE AN UNDERLYING INFECTIVE AETIOLOGY.

E. A. FIRTH AND F. J. C. ROE.

*From the Cancer Research Department, London Hospital Medical College, London, E.*1.

Received for publication April 28, 1955.

It is well known that in many breeds of mice pulmonary adenomata occur spontaneously (Law, 1954). In some breeds the incidence may approach 100 per cent in older mice although the number of tumours borne by individual mice is usually low. Several of the carcinogenic hydrocarbons, appropriately administered increase the incidence of pulmonary adenomata in such susceptible strains of mice (Andervont and Shimkin, 1940). Gamma rays also induce the formation of these tumours (Lorenz *et al.*, 1946), and Rogers (1955) reported that adenomata develop in grafts of foetal lung previously irradiated *in vitro* with ultraviolet light. There are breeds, however, which have no natural incidence of pulmonary tumours, and in these it may be difficult or impossible to induce lung tumour-formation by chemical agents (Gross *et al.*, 1953).

The genetics of pulmonary tumours was first studied by Lynch (1926). Law (1954) reviews the now considerable literature on this subject. He concludes that susceptibility to lung tumours (both spontaneous and induced) is influenced by genes located on at least four chromosomes ; and a relationship has been.demonstrated (Heston *et al.*, 1952) between genes which lower the incidence of lung tumours and those which decrease body weight.

Nettleship and Henshaw (1943) were the first to describe the high incidence of pulmonary tumours in mice after treatment with urethane (ethyl carbamate). This finding, now amply confirmed, has been studied by many other workers. The induction of lung tumours by urethane does not depend on any particular route of administration. Intraperitoneal injection (Nettleship and Henshaw, 1943), oral administration (Selbie and Thackray, 1948), and application to the skin (Cowen, 1950) have all been shown to be effective. Moreover, induction by the transplacental route has been demonstrated : urethane administered to the pregnant female of a susceptible strain leads to a high incidence of lung tumours in the progeny (Larsen, 1947*a*).

Larsen (1947*b*, 1948) tested a series of carbamates related to urethane, and a number of alkylated urethanes. Ethyl N-isopropyl carbamate was found to possess moderate lung tumour-inducing activity, but none of the other compounds examined showed more than slight activity. Larsen (1950) also tested some of the possible breakdown-products of urethane, namely ethyl alcohol, carbon dioxide (in the form of sodium bicarbonate), ammonia (as ammonium carbonate and ammonium chloride), and cyanic acid (as potassium cyanate). He concluded that the effect observed following the administration of urethane was most probably due to urethane itself and not to one of its breakdown products, and that within the series of related compounds tested the activity was almost specific for urethane.

Urethane possesses both antileukaemic and narcotic properties, and several substances which possess one of these properties have been tested for lung tumour-inducing action. Larsen, Rhoads and Weed (1946) examined a number of narcotic agents, but none showed activity. Shimkin (1954) tested a series of antileukaemic agents. He reported that apart from urethane both nitrogen mustard (HN2) and triethylene melamine (T.E.M.) were capable of increasing the incidence of lung tumours in mice. Aminopterin, Myleran, stilbamidine, Fowler's solution, and cortisone all failed to do so.

Despite the simplicity of the urethane molecule, the chemical mechanism(s) underlying its various pharmacological actions is almost a complete mystery. Todd (quoted by Haddow and Sexton, 1946) suggested that urethane may interfere with nucleotide synthesis. In support of this Cowen (1949) demonstrated that the induction of lung tumours by urethane was inhibited by the simultaneous administration of pentose nucleotides. Boyland (1952) thought that urethane might cause thymine-deficiency by prevention of the methylation of uracil to thymine. Later (Boyland and Koller, 1954), it was found that simultaneous administration of thymine reduced the incidence of abnormal mitosis due to urethane in the Walker rat carcinoma 256. Haddow and Sexton (1946) suggested that urethane may interfere with purine synthesis. There is evidence (Roe, 1955) that simultaneous administration of the purine precursors, glycine and formate, inhibits the initiation of skin tumour-formation by urethane (Salaman and Roe, 1953).

The observations of Noble and Millar (1948) cannot be fitted into the general background of work, briefly reviewed above, on the genetics and chemical induction of pulmonary tumours and, in particular, on the chemical basis of the action of urethane. These workers, in a preliminary report, recorded the occurrence of adenomata in the lungs of a small number of " Swiss " albino mice 6 months after the subcutaneous injection of a suspension of adenoma-containing lung tissue, taken from donor mice which had been treated with urethane not less than 5 months before. In another experiment mice were injected with lung tissue from donor mice which had been given injections of urethane 3, 6, or 24 hours previously. Single or multiple adenomata were seen in the lungs of the recipient mice at *post mortem* 6 months later. Control mice injected with lung tissue from untreated donors developed no tumours. It seems unlikely that sufficient urethane could have persisted in the donor lung to account for the subsequent development of lung adenomata in the hosts, in view of the findings of Bryan, Skipper and White (1949), Skipper *et al.* (1951), and Boyland and Rhoden (1949) who showed that at least 90 per cent of urethane injected into normal mice is metabolised in the first 24 hours after injection.

The experiment described below was designed as a repetition of the work of Noble and Millar (1948). A repetition was clearly desirable because of the difficulty of reconciling their results with the most generally held view of the aetiology of lung adenomata, namely that their occurrence depends on two factors only, a genetic susceptibility, and the action of extraneous chemical or physical agents. It has been suggested however (Selbie and Thackray, 1948) that an infective agent may also play a part in the production of lung adenomata in mice. A confirmation of Noble and Millar's results would strengthen this suggestion.

### EXPERIMENTAL.

Male mice of the stock albino strain " S " (Salaman and Gwynn, 1951), aged approximately 8–10 weeks were treated as follows :

(*a*) Fifteen mice were injected subcutaneously, and a further 8 mice intraperitoneally, with a suspension of adenoma-bearing lung tissue from mice previously treated with urethane [3 to 18 applications of 0·3 ml. of a 20 per cent w/v solution of urethane in acetone (0·18 to 1·08 g.) to the skin]. Each recipient mouse was given the equivalent of one lung from a donor mouse, consisting of up to 50 per cent of adenomatous tissue. Before injection the lung was homogenized and suspended in Ringer solution. None of the donor mice had received treatment with urethane for at least 10 weeks before sacrifice.

Thirteen mice injected subcutaneously and 5 intraperitoneally survived until 6 months after injection. Of these 16 had no lung adenomata, 2 others bore one adenoma each. This incidence is well within the limits for untreated mice of the same age and strain.

(*b*) To control against a possible positive result in the above experiment being due to residual urethane in the injected lung tissue, three groups, each of 4 mice, were injected with the equivalent of one lung from donor mice which had received two injections of 20 mg. urethane in 0·4 ml. Ringer solution intraperitoneally 24 and 4 hours, 72 and 48 hours, and 8 and 7 days, respectively, before sacrifice. The lung homogenate was heated in a water bath at 100° C. for 15 minutes, before injection, in order to destroy any heat-sensitive agent while preserving unmetabolised urethane. (In a preliminary experiment it was shown that similar heat treatment of an aqueous solution of urethane (100° C. for 15 minutes) caused no loss of anaesthetic potency.)

A further group of 4 mice were injected intraperitoneally with heat-treated homogenized liver (equivalent of half a liver per recipient mouse) from donor mice injected twice with 20 mg. urethane (24 and 4 hours previously).

Six months later the 14 survivors of this experiment were killed, and *post mortem* examination revealed no adenomata in 12 of the mice, and one adenoma in each of the remaining 2. This incidence was well within the limits for untreated mice.

### CONCLUSION.

These experiments, essentially similar to those of Noble and Millar (1948), but conducted in a different strain of mice, failed to confirm their results. The suggestion that an infective agent is concerned in the production of the lung adenomata which develop after urethane treatment is not supported by the present findings.

### SUMMARY.

1. The experiments of Noble and Millar (1948), in which lung adenomata developed in mice injected with suspensions of adenomatous lung obtained from urethane-treated mice, were repeated in a different strain. Their results were not confirmed.

2. Noble and Millar's findings had suggested that an infective agent was concerned in the development of the lung adenomata which follow urethane treatment. This and other reports on the problem are discussed. It is concluded that no unequivocal evidence for an infective aetiology exists at present.

ADDENDUM.

During the preparation of this paper Professor R. L. Noble kindly communicated to us the results of experiments carried out subsequent to the publication of the preliminary report referred to in the text (Noble and Millar, 1948).

In the course of a repetition of their original experiments with much larger numbers of animals it was found that the " Swiss " albino strain used had a high incidence of spontaneous lung adenomata. None of the experimental procedures originally used increased this incidence significantly.

We thank Dr. M. H. Salaman for his advice. The expenses of this research were partly defrayed out of a Block Grant from the British Empire Cancer Campaign.

REFERENCES.

ANDERVONT, H. B. AND SHIMKIN, M. B.—(1940) *J. nat. Cancer Inst.*, **1**, 225.
BOYLAND, E.—(1952) *Cancer Res.*, **12**, 77.
*Idem* AND KOLLER, P. C.—(1954) *Brit. J. Cancer*, **8**, 677.
*Idem* AND RHODEN, E.—(1949) *Biochem. J.*, **44**, 528.
BRYAN, C. E., SKIPPER, H. E. AND WHITE, L.—(1949) *J. biol. Chem.*, **177**, 941.
COWEN, P. N.—(1949) *Brit. J. Cancer*, **3**, 94.—(1950) *Ibid.*, **4**, 337.
GROSS, L., GLUCKMAN, E. C., KERSHAW, B. B. AND POSSELT, A. E.—(1953) *Cancer*, **6**, 1241.
HADDOW, A. AND SEXTON, W. A.—(1946) *Nature*, **157**, 500.
HESTON, W. E., DERINGER, M. K., HUGHES, I. R. AND CORNFIELD, J.—(1952) *J. nat. Cancer Inst.*, **12**, 1141.
LARSEN, C. D.—(1947*a*) *Ibid.*, **8**, 63.—(1947*b*) *Ibid.*, **8**, 99.—(1948) *Ibid.*, **9**, 35.—(1950) *Cancer Res.*, **10**, 230.
*Idem*, RHOADS, P. B. AND WEED, L. L.—(1946) *J. nat. Cancer Inst.*, **7**, 5.
LAW, L. W.—(1954) 'Advances in Cancer Research.' New York (Academic Press Inc.), II, pp. 298–304.
LORENZ, E., HESTON, W. E., DERINGER, M. K. AND ESCHENBRENNER, A. B.—(1946) *J. nat. Cancer Inst.*, **6**, 349.
LYNCH, C. J.—(1926) *J. exp. Med.*, **43**, 339.
NETTLESHIP, A. AND HENSHAW, P. S.—(1943) *J. nat. Cancer Inst.*, **4**, 309.
NOBLE, R. L. AND MILLAR, M. J.—(1948) *Nature*, **162**, 253.
ROE, F. J. C.—(1955) *Ibid.*, **175**, 636.
ROGERS, S.—(1955) *J. nat. Cancer Inst.*, **15**, 1001.
SALAMAN, M. H. AND GWYNN, R. H.—(1951) *Brit. J. Cancer*, **5**, 252.
*Idem* AND ROE, F. J. C.—(1953) *Ibid.*, **7**, 472.
SELBIE, F. R. AND THACKRAY, A. C.—(1948) *Ibid.*, **2**, 380.
SHIMKIN, M. B.—(1954) *Cancer*, **7**, 410.
SKIPPER, H. E., BENNETT, L. L., BRYAN, C. E., WHITE, L., NEWTON, M. A. AND SIMPSON, L.—(1951) *Cancer Res.*, **11**, 46.